# Cardiovascular Activities of of the bradykinin system

**DOI:** 10.1100/tsw.2008.53

**Published:** 2008-04-14

**Authors:** Jagdish N. Sharma

**Affiliations:** Department of Applied Therapeutics, Faculty of Pharmacy, Health Sciences Center, Kuwait University, P.O. Box 24923, Safat 13110, Kuwait

**Keywords:** kallikrein-kinin system, cardioprotection, hypertension, cardiac diseases, angiotensin-converting enzyme inhibitors

## Abstract

All the components of the kallikrein-kinin system are located in the cardiac muscle andits deficiency may lead to cardiac dysfunction. In recent years, numerous observationsobtained from clinical and experimental models of diabetes, hypertension, cardiacfailure, ischemia, myocardial infarction, and left ventricular hypertrophy have suggestedthat the reduced activity of the local kallikrein-kinin system may be instrumental for theinduction of cardiovascular-related diseases. The cardioprotective property of theangiotensin-converting enzyme inhibitors is primarily mediated via a kinin-releasingpathway, which may cause regression of the left ventricular hypertrophy in hypertensivesituations. The ability of kallikrein gene delivery to produce a wide spectrum of beneficialeffects makes it a promising candidate in treating hypertension and cardiovascular andrenal diseases. In addition, stable kinin agonists may also be available in the future astherapeutic agents for cardiovascular and renal disorders. However, there are alsopossibilities of adverse effects that may be caused by these compounds.

## INTRODUCTION

A number of observations focus on the kinins as potential mediators in endogenous, cardiovascular protective mechanisms. This is due to the fact that kallikrein-kinin system (KKS) components are localized in the heart and in the vascular tissues[1,2,3,4,5,6]. Kinins are released during ischemia[7] and cause beneficial cardiac effects[8]. Bradykinin (BK) antagonists worsen ischemia-induced effects[9] and BK can contribute to the cardioprotective effects of preconditioning[10]. On the other hand, the reduction in cardiac infarct size by BK, after preconditioning in rabbits, was prevented by a BK antagonist (Hoe 140) treatment[10]. BK at a dose that has no effect on blood pressure (BP) can prevent left ventricular hypertrophy (LVH) in rats with hypertension caused by aortic banding[11]. Reduction in peripheral and cardiac KKS components may also be the cause of developing high BP in human and experimental animals[12,13,14,15]. In the present review, the current concept on the role of kinins in the cardiovascular system is presented.

## THE KININ SYSTEM

The kinins are pharmacologically active polypeptides, which are released in the tissues and body fluids as a result if the enzymatic action of kallikreins on kininogens. The kinin family includes BK (Arg-Pro-Pro-Gly-Phe-Ser-Pro-Phe-Arg), kallidin (Lys-Arg-Pro-Pro-Gly-Phe-Ser-Pro-Phe-Arg), and methionyl-lysyl-BK (Met-Lys-Arg-Pro-Pro-Gly-Phe-Arg). Kallidin and methionyl-lysyl-BK are converted into BK by aminopeptidases present in plasma and urine[16]. Kinins are rapidly (<15 sec) inactivated by circulating kininases[17].

Kininogens are multifunctional proteins derived mainly from alpha-2 globulin. In humans, the two forms of kininogens are high-molecular-weight kininogen (HMWK) and low-molecular-weight kininogen (LMWK)[18].

These kininogens vary from each other in molecular weight, susceptibility to plasma and tissue kallikreins, and in their physiological properties[19]. They are synthesized in the liver and circulate in the plasma and other body fluids. In addition, there is a T-kininogen in rat plasma, which is considered to be an acute-phase reactant of inflammation[20]. This kininogen releases T-kinin by the enzymatic action of T-kallikrein in rats[21]. Tissue kallikrein is found in various organs, such as the kidney, heart, and synovial tissue[1,2,3,22,23]. These kallikreins differ from one another in molecular weight, biological function, and physicochemical and immunological properties[24]. The tissue kallikrein is synthesized in the cells as a precursor and converted into active form by the cleavage of an amino terminal peptide[25]. Active tissue kallikrein acts on LMWK to release kallidin. The plasma kallikrein is found in circulation in an inactive form, which is known as prekallikrein or Fletcher factor[26]. This inactive prekallikrein is converted to active kallikrein by activated Hageman factor (XIIa)[27]. In addition, plasma kallikrein is able to convert inactive factor XII to XIIa by positive feedback reaction. The plasma prekallikrein and HMWK are present together in a complex form[27]. Factor XIIa and factor XI circulate with HMWK in bound form[29]. In this way, factor XI can be converted into XIa for the participation in the intrinsic coagulation cascade[30]. In immunological reactions, the tissue proteoglycone and mast cell heparin might act as an initiating surface for initial activation of the Hageman factor[31]. It seems that the kinins may be generated in parallel with the formation of thrombin at inflammatory sites, since inactive plasma kallikrein can be activated by coagulant Hageman factor. The tissue kallikrein multigene family comprises a closely related cluster of genes that vary in number between the different mammalian species: 24 genes have been identified in the mouse, 20 in the rat, three in humans, and three in the hamster[24].

Several restriction fragment length polymorphisms (RFLP) have been mapped in the tissue kallikrein gene and their regulatory regions in spontaneously hypertensive rats (SHR)[32]. These findings may reflect a possible difference in the tissue kallikrein gene locus between SHR and normotensive Wistar-Kyoto rats (WKYR). A tissue kallikrein RFLP has been indicated to cosegregate with high BP in the F2 offspring of SHR and normotensive Brown Norway rats crosses[33]. This finding strongly suggests a possibility of SHR. The kininases, kinin inactivating enzymes, are present in the plasma, endothelial cells, and in the tissues to regulate the physiological functions of the kinins in the body. These are known as kininase I, kininase II or angiotensin-converting enzyme (ACE), and enkaphalinase. In plasma, kininase I cleaves the C-terminal arginine of BK to form des-Arg9-BK[34]. Kininase II causes inactivation of BK by releasing pentapeptide (Arg-Pro-Pro-Gly-Phe) and tripeptide (Ser-Pro-Phe) fragments. Fig. 1 shows the kinin formation, activation, and inhibition pathways.

## KININ RECEPTORS AND ANTAGONISTS

Kinins exert their pharmacological actions through the activation of two receptor types, B1 and B2, which have been cloned and belong to the seven transmembrane G-protein coupled receptor families[35]. The kinin B1 receptor displays high affinity and selectivity for kinin metabolites lacking the C-terminal arginine residue, such as des-Arg9-BK. The B1 receptor is rarely expressed in normal tissue, but seems to

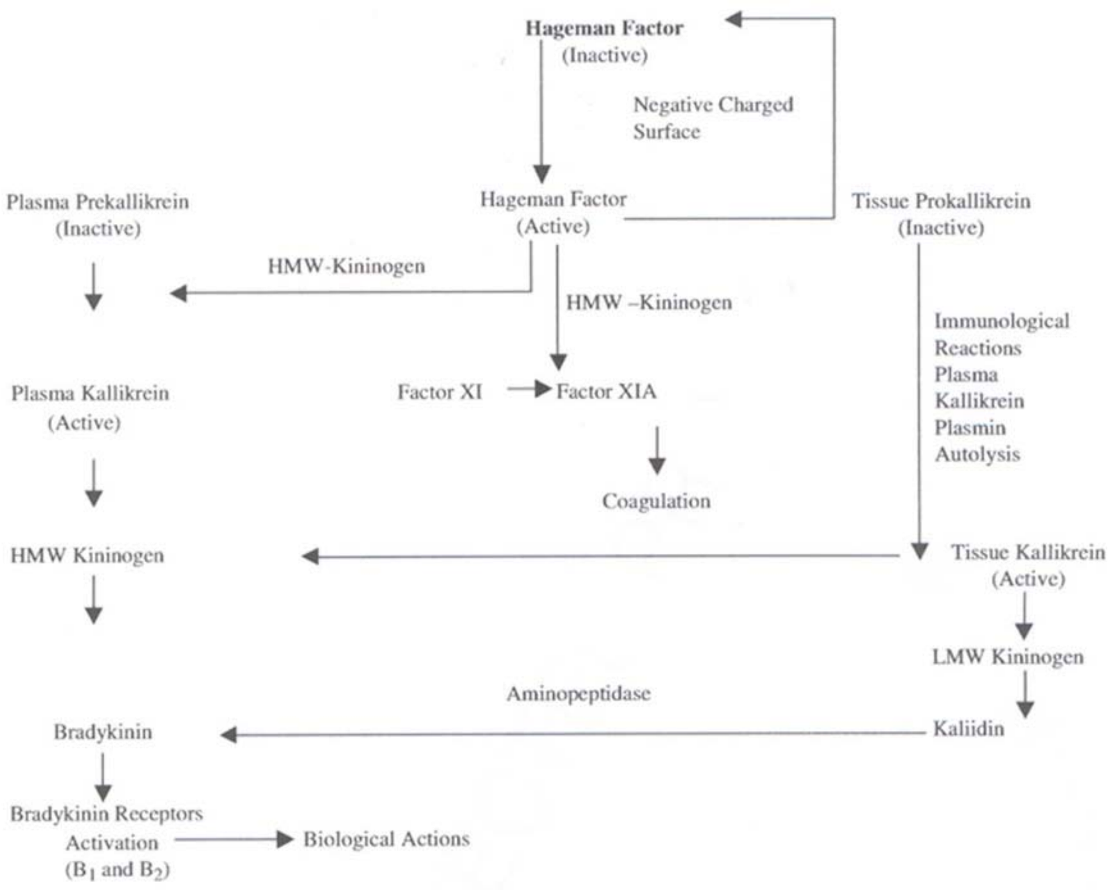

**FIGURE 1.** The mode of kinin formation.

be up-regulated in pathological states associated with inflammation and tissue injury[35]. This may indicate an important area of research within the study of KKS. B1 receptor activation may produce stimulation of smooth muscle, increased cell proliferation, and collagen synthesis[36]. In addition, it may also the cause release of nitric oxide (NO) and prostacyclin ($PGI_2$) from bovine endothelial cells[37]. Kinins stimulate the release of tumor necrosis factor and interleukin from macrophages through activating B1 receptors[38]. The kinin B2 receptors may participate in pathological conditions, such as pain[39], inflammation[40,41], bronchoconstriction[42], hypertension[43], and cardiac arrhythmias induced in rats[44,45]. The B2 receptor is thought to mediate contractions of rat uterus, guinea-pig ileum, and tracheal smooth muscles[46]. Kinins act on kinin B2 receptors to release conjointly NO and $PGI_2$ from the endothelial cells *in vitro*[37]. B2 receptors exhibit higher affinity for BK and kallidin. Farmer et al.[47] suggested that the large airways contain a novel B3 receptor, which may produce BK-induced bronchoconstriction. These investigators noted that several B2 receptor antagonists, such as D-Arg (Hyp3,D-Phe-7)-BK and D-Arg(Hyp3, Thi5,8,D-Phe7)-BK, as well as B1 antagonist (des-Arg9 [Leu8]-BK), did not block the BK-induced contraction of guinea-pig tracheal smooth muscle preparations. The presence of a kinin B3 receptor has also been proposed in the opossum esophageal longitudinal smooth muscle[48]. This receptor has been characterized by rapid desensitization, contraction of longitudinal smooth muscle via PG release, and activation by kinin B2 receptor antagonists (Phe8-D-Phe7-BK and D-Phe7-hyp8-BK). Furthermore, Saha et al.[49] proposed the presence of a B4 receptor in the opossum esophageal longitudinal smooth muscle. This receptor shows no tachyphylaxis; its action does not involve PG and it is activated by kinin B2 receptor antagonists (Thi5, 8-D-Phe7, and B6572). The development of kinin receptor antagonists has been pursued for more than 2 decades[50,51]. The kinin B1 receptor antagonist was first introduced as des-Arg9-(leu8)-BK by Regoli and Barabe[50]. The "second generation" of B2 receptor antagonist came with the introduction of Hoe 140 (D-Arg-Arg-Pro-Hyp-Gly-Thi-Ser-D-Tic-Ag; Icatibant)[52] and CP-0127 (B(dArg-Arg-Pro-Hyp-Gly-Phe-Cys-DPhe-Leu-Arg)2[53]. The "third generation" of BK antagonist, B9430 (DArg-Arg-Pro-Hyp-Gly-Igl-Ser-DIgl-Oic-Arg) is known to be extremely potent and long lasting at both B1 and B2 receptors[54]. The development of this compound not only demonstrates that a polypharmaceutic approach covering both receptor types is possible, but also that the structures of the B1 and B2 receptors are sufficiently similar to be antagonized by a single drug. This fact was not appreciated until recently. Most recently, bradyzide, a potent nonpeptide B2 BK receptor with long-lasting oral activity in animal models of inflammatory hyperalgesia, has been described[55]. These BK receptor antagonists may prove to be therapeutically applicable in pathological states, which are caused by hyperactivity of kinins.

## MODE OF THE KININ ACTION

Interaction between the kinins and their specific receptors can lead to activation of several second-messenger systems. The kinin receptor stimulation in the intact cells or in tissues appear to initiate the second-messenger pathways, such as arachidonic acid products and the activation of calcium-sensitive systems[56]. The elevation of cellular inositol phosphates by BK involves G-protein coupled activation of phospholipase A2 and C that are used in the synthesis of eicosanoids[57]. It is of interest that indomethacin, a cyclooxygenase inhibitor, was able to cause potentiation of BK-induced contractions of both isolated estrous rat uterus and guinea-pig tracheal smooth muscle preparations[58,59]. These findings may suggest that there could be noneicosanoid pathways for the cellular and molecular actions of BK. Furthermore, it is known that BK significantly stimulates phosphoinositide hydrolysis in guinea-pig ileum longitudinal muscle that may result in elevation of cytosolic calcium ion levels to induce contractile responses[60]. Fischer et al.[61] demonstrated that the kinin B2 receptor stimulation causes production of cyclic guanosine monophosphate (cyclic GMP) in cultured porcine aortic endothelial cells. The formation of cyclic GMP may be an important step for the biological actions as well as release of NO evoked by BK in the endothelial cells and in the vascular smooth muscles.

## THE KININ SYSTEM IN CARDIOVASCULAR DISORDERS

### Hypertension

Hypertension is a major risk factor for the development of cardiovascular diseases, such as coronary heart disease, congestive heart failure, and peripheral vascular and renal diseases[13]. There is ample evidence documenting the role of KKS in the pathogenesis of hypertension[15]. The pharmacological action of BK in the regulation of systemic BP was vasodilatation in most areas of the circulation, a reduction of total peripheral vascular resistance, and a regulation of sodium excretion from the kidney[62,63]. When BK is injected into the renal artery, it causes diuresis and natriuresis by increasing renal blood flow[64]. These actions of BK have been attributed to prostaglandin release in the renal circulation[65]. The role of KKS in hypertension was established by Margolius and coinvestigators[65,66] with the observations that urinary kallikrein excretion is significantly reduced in hypertensive patients and hypertensive rats. This led to the suggestion that reduced urinary kallikrein excretion might result from a defect in kinin generation in hypertensive situations. Research on the systemic changes in the KKS has provided further insight regarding the mechanisms of various hypertensive conditions. In this connection, it is known that kininogen levels and a kinin-potentiating factor are reduced in essential and malignment hypertension[67,68,69,70]. It may be possible that the deficiency in plasma HMWK is due to decreased liver synthesis in individuals who develop hypertension after mild exercise[71]. It can be proposed that a deficient KKS might be a significant factor in the pathophysiology of hypertension. In this connection, it is suggested that the role of renal KKS is to excrete excess of sodium. Therefore, a reduction in the generation of renal KKS may be the cause of the development of hypertension as a result of sodium accumulation in the body[72,73]. Thus, the development of a compound having renal kallikrein-like activity may serve the purpose of excreting excessive sodium from the kidney. This action may be useful for the treatment of hypertension. Also, it has been demonstrated that transgenic mice that overexpress renal tissue kallikrein were hypotensive and that the administration of aprotinin, a tissue kallikrein inhibitor, restored the BP in the transgenic mice[74]. The suppression of the hypotensive responses of ACE inhibitors by aprotinin in SHR has been documented[75]. These findings highlight a role of tissue kallikrein in the regulation of BP. Recently, it has been proposed that tissue kallikrein gene delivery into various hypertensive models exhibits protection, such as a reduction in high BP, attenuation of cardiac hypertrophy, inhibition of renal damage, and stenosis[76]. These findings may indicate the prospect of this kallikrein gene therapy for cardiovascular and renal pathology. Kininase II (ACE) inhibitors are currently used in the treatment of both clinical and experimental hypertension[77,78,79]. Kininase II inhibitors could lower BP by inhibiting the biodegradation of kinin as well as blocking the formation of angiotension II (Ang II) at the renal site. A calcium-channel blocker, nifedipine, used to treat patients with essential hypertension, can normalize the reduced urinary kallikrein excretion[80]. Our previous investigations demonstrated differential sensitivity for the genetically Dahl-salt-sensitive (DSS) hypertensive and genetically Dahl-salt-resistant (DSR) normotensive rats to the hypotensive action on nifedipine[79]. This might reflect a significantly more important function of diminished renal KKS activity in DSS hypertensive, as compared with the DSR normotensive, rats. It is unknown whether a similar situation may exist in genetically predisposed humans with hypertension. Furthermore, Smith et al.[80] have proposed that women with reduced activity of the renal KKS combined with increased sympathetic drive may be at increased risk of developing pregnancy-induced hypertension. It is a generally accepted view that the BK-induced BP lowering effect is mediated by the kinin B2 receptor, but B1 might also be involved under special situations[36]. It has been demonstrated that the B2 receptor antagonist (B5630) can abolish the hypotensive effects of BK as well as captopril, an ACE inhibitor[43]. This led to the proposal that the hypotensive action of ACE inhibitors might be due to the activation of the kinin B2 receptor[81,82]. The accumulation of BK after treatment with ACE inhibitors with subsequent release of NO, prostaglandins, and $PGI_2$ could account for the additional mediators released by these drugs in hypertensive patients. However, the use of BK antagonists can abolish the effectiveness of antihypertensive drugs; therefore, these drugs must be contraindicated in patients with hypertension.

## Cardiac Failure and Ischemia

Cardiac failure and ischemia are the leading cause of death in the developed and many developing countries[83]. These conditions are considered as the new emerging epidemic of the third millennium[83]. The role of kinins in the heart did not receive much attention, despite the fact that it was shown earlier[84] that local and systemic administration of BK can increase coronary blood flow and improve myocardial metabolism. It is well known that ACE inhibitors limit ventricular dilatation, delay the progression of clinical symptoms, and improve mortality rate. These beneficial actions appear to be related to the reduced formation of Ang II, which results in a decreased growth response and attenuated pressure load[84]. In addition, the ability of ACE inhibitors to prevent kinins from enzymatic breakdown represents a relevant mechanism contributing to cardioprotection[85]. This concept fueled a series of studies demonstrating the presence of a local KKS in the heart[1,2,4]. The binding of kinins to endothelial B2 receptors leads to the release of NO and $PGI_2$, exerting vasodilator, ischemic, antiproliferative effects and preserving myocardial stores of energy-rich phosphates and glycogen[86]. Kinins contribute to the maintenance of cardiovascular homeostasis by opposing the vasoconstrictor activity of Ang II[87]. Circumstantial evidence also suggests that a dysfunctional KKS may contribute to the pathogenesis of heart failure. In fact, reduced local kinin generation and blunted NO formation have been reported in microvessels of failing human hearts[88]. Furthermore, in dogs with pacing-induced congestive heart failure, selective blockade of B2 receptors by Hoe 140 reduces coronary blood flow and contractility, and increases left ventricular end diastolic pressure[89]. Thus, the reduced activity of the cardiac KKS may facilitate the development of cardiac failure. On the other hand, kinins are continuously released during cardiac hypoxia and ischemia[8,90]. They act as cardioprotective agents in perfusion and participate in the process of ischemic preconditioning[7,10]. There is evidence to suggest that BK infusion into coronary artery reduces significantly the severity of ischemia-induced arrhythmia in anesthetized dogs[90]. Studies undertaken in rats, dogs, and humans revealed that kinins are released under the conditions of ischemia and myocardial infarction[91,92,93,94]. This process may be an indicator of the role of kinin in protecting the heart at the time of myocardial infarction. This raised local kinin release might be able to exert a protective effect on the heart by activating signal transduction pathways generating NO and $PGI_2$. Coronary artery ligation for shorter and longer duration in SHR and WKYR showed that administration of BK could increase the survival time of these rats[44,45]. This effect of BK was reverted by pretreatment with a specific B2 receptor antagonist[45]. In conclusion, these results support the hypothesis that KKS might be regarded as a prime mediator in protecting the heart in ischemic conditions. However, extensive investigations on the molecular biology and gene mapping of KKS in the heart during health and cardiovascular diseases can provide many questions to be answered regarding the significance of KKS in cardiovascular pathophysiology. This may allow us to develop KKS-based therapeutics for the cardiovascular diseases.

## Left Ventricular Hypertrophy

Left ventricular hypertrophy (LVH) is regarded as an independent risk factor in hypertensive patients[3]. BK can counter the development of LVH in rats with hypertension produced by aortic banding[11]. This antihypertrophic effect of BK was abolished by treatment with B2 receptor antagonist and NO synthetase inhibitor. Thus, BK has a role in protecting the heart against developing LVH by releasing NO in this model of hypertension induced by aortic banding. In this regard, we have demonstrated for the first time that a lack of the cardiac KKS could be responsible for the induction of LVH in SHR and SHR with diabetes[2,3,4]. Therefore, it is suggested that the reduced cardiac tissue kallikrein and cardiac kininogen may be responsible for reduced BK generation in the heart. Therefore, deficient components of the KKS in the heart may be the cause of myocardial dysfunction in maintaining high BP and cardiac LVH. It is highly desired the stable compounds of KKS be developed in order to evaluate their efficacy and potency in cardiac failure, cardiac ischemia, as well as myocardial infarction. Recently, we have shown that, in hypertensive rats, BP reduction and regression of LVH with captopril treatment might be due to enhanced renal tissue kallikrein activity[93]. This may further support the view that tissue kallikrein may act as a cardioprotective agent. It has been recently proposed that kinins have modulatory effects in preventing myocardium ischemia[95,96]. It is of interest to note that Madeddu and coworkers[97] described the cardiac hypertrophy and microvascular deficit in kinin B2 receptor knockout mice. The different synergistic cardioprotective actions of the BK in the abnormal diabetic heart may indicate that the stimulation of the BK system might be a useful tool for treating cardiopathy caused by diabetes[98]. A recent study conducted in diabetic and hypertensive rats has proposed that higher plasma prekallikrein levels may be an indicator for predicting hypertension and LVH[99]. Furthermore, activation of BK2 receptor may promote antioxidant, anti-inflammatory properties in protecting against stroke, cardiovascular and renal diseases, and may serve as new drug targets for preventing and treating heart failure, vascular injury, end-stage renal disorders, and stroke in humans[100].

## CONCLUSION

The evidence presented in this review suggests that the KKS has a greater role to play in the various pathophysiological processes of the cardiovascular system, such as hypertension, cardiac failure and ischemia, LVH, and endotoxemia. There is activation of BK activity in endotoxemia. Under this situation, the inhibition of KKS activities by the application of the kallikrein inhibitors, B1 and/or B2 receptor antagonists, may be able to reverse the pathological consequences. It is of interest to state that there is the possibility of the up-regulation of the B1 and B2 receptors in these pathological conditions. On the other hand, it seems that there is deficient activity of the KKS in the pathological conditions of hypertension, cardiac ischemia, and development pf LVH. These pathological states may be due to genetic abnormality of the KKS or down-regulation of BK receptors. These diseases may be treated with the application of tissue kallikrein and/or use of specific BK receptor agonists. Furthermore, the mode of cardioprotective effect of ACE inhibitors might be mediated via KKS.
